# *ANLN* and *TLE2* in Muscle Invasive Bladder Cancer: A Functional and Clinical Evaluation Based on In Silico and In Vitro Data

**DOI:** 10.3390/cancers11121840

**Published:** 2019-11-21

**Authors:** Sheng Wu, Katja Nitschke, Jakob Heinkele, Cleo-Aron Weis, Thomas Stefan Worst, Markus Eckstein, Stefan Porubsky, Philipp Erben

**Affiliations:** 1 Department of Urology and Urosurgery, Medical Faculty Mannheim, University of Heidelberg, 68167 Mannheim, Germany; sheng.wu@medma.uni-heidelberg.de (S.W.); katja.nitschke@medma.uni-heidelberg.de (K.N.); jakob.heinkele@medma.uni-heidelberg.de (J.H.); thomas.worst@medma.uni-heidelberg.de (T.S.W.); 2 Institute of Pathology, Medical Faculty Mannheim, University of Heidelberg, 68167 Mannheim, Germany; cleo-aron.weis@umm.de (C.-A.W.); stefan.porubsky@umm.de (S.P.); 3 Institute of Pathology, University Hospital Erlangen, Friedrich-Alexander-University Erlangen-Nürnberg, 91052 Erlangen, Germany; markus.eckstein@uk-erlangen.de

**Keywords:** muscle invasive bladder cancer, prognosis, biomarker, molecular subtype

## Abstract

Anilin actin binding protein (ANLN) and transducing-like enhancer protein 2 (TLE2) are associated with cancer patient survival and progression. The impact of their gene expression on progression-free survival (PFS) of patients with muscle invasive bladder cancer (MIBC) treated with radical cystectomy (RC) and subtype association has not yet been investigated. qRT-PCR was used to measure the transcript levels of *ANLN* and *TLE2* in the Mannheim cohort, and validated in silico by The Cancer Genome Atlas (TCGA) cohort. Uni- and multivariate Cox regression analyses identified predictors for disease-specific survival (DSS) and overall survival (OS). In the Mannheim cohort, tumors with high *ANLN* expression were associated with lower OS and DSS, while high *TLE2* expression was associated with a favorable OS. The TCGA cohort confirmed that high *ANLN* and low *TLE2* expression was associated with shorter OS and disease-free survival (DFS). In both cohorts, multivariate analyses showed *ANLN* and *TLE2* expression as independent outcome predictors. Furthermore, *ANLN* was more highly expressed in cell lines and patients with the basal subtype, while *TLE2* expression was higher in cell lines and patients with the luminal subtype. *ANLN* and *TLE2* are promising biomarkers for individualized bladder cancer therapy including cancer subclassification and informed MIBC prognosis.

## 1. Introduction

Bladder urothelial carcinoma (BLCA) is the most common urinary tumor worldwide with about 386,000 new cases and nearly 150,200 deaths each year [1]. Non-muscle-invasive bladder cancers (NMIBC; 70%) are not immediately life threatening but often progress, while muscle-invasive bladder cancers (MIBC; 30%) are responsible for the most cases of metastases and death [2]. The current therapeutic standard for MIBC is radical cystectomy (RC) with perioperative platinum-based chemotherapy in selected cases [3]. The clinical management of MIBC is currently limited. First, potentially inadequate treatment decisions are informed by a limited clinicopathological staging system [4]. Therefore, mRNA-based molecular subtyping of MIBC, which shows target genes enriched in specific molecular subtypes, has emerged as a promising tool with the potential to stratify patients for treatment [5]. Luminal and basal differentiated tumors are characterized by specific gene expression, such as *Keratin 5* (*KRT5*) in the basal subtype and *KRT20* in the luminal subtype [6]. Second, the onset of BLCA is a multi-factorial and multi-step formation process involving multiple genes [7,8]. Therefore, in-depth study of the molecular mechanisms of the genes closely related to the malignant progression of BLCA and therapeutic relevant targets will help to understand the regulatory mechanisms of key genes in the development and progression of BLCA.

*Anilin actin binding protein* (*ANLN*) is located on chromosome 7q14.2 and encodes for a 1,125 amino acid actin-binding protein that includes a conserved N-terminal actin (F-actin) and myosin binding region and a conserved C-terminal pH binding domain [9]. Previous studies found that *ANLN* expression levels were significantly up-regulated in a variety of tumor tissues, including breast, ovarian, colon, lung, and pancreatic cancers [10,11,12,13,14,15]. Furthermore, *ANLN* and its encoded protein are highly expressed in BLCA tissues, and their expression levels are positively correlated with the pathological grade and stage of BLCA [16]. However, the involved signaling pathways and the interacting molecular targets in the regulation of BLCA biological function are in discussion.

*Transducing-like enhancer protein 2* (*TLE2*), a member of the TLE gene family, is located on chromosome 19p13.3, and acts as a transcriptional corepressor [17]. Previous studies revealed that the molecular properties of the *TLE2* protein point to a function in transcriptional regulation, involved in embryonic neuronal development in conjunction with Hairy/Enhancer of split (HES) proteins [17,18]. The *TLE2* protein inhibits replication-and-transcription-activator-mediated transactivation and lytic reactivation of Kaposi’s sarcoma-associated herpesvirus [19]. The function of *TLE2* in BLCA has not yet been investigated.

In a previous study, *ANLN* expression was associated with Wnt/β-catenin signaling in gastric cancer [20]. Also, the Wnt/β-catenin pathway regulates gene expression via T-cell factor/lymphoid enhancer-binding factor 1 (TCF/LEF1) family, which is repressed by transcription factors for TLEs [21,22]. However, the association between *ANLN* and *TLE2* was not elucidated yet.

This study was the first to investigate the expression of *TLE2* in human urothelial cell lines and MIBC samples in correlation with histopathology and survival data. The involved signaling pathways and the impact of *ANLN* and *TLE2* on MIBC molecular subtypes were also evaluated.

## 2. Results

### 2.1. Patient Population and Survival Analysis

Demographic and clinical-pathological data of the 60 patients with MIBC included in the Mannheim cohort are shown in Table 1. Median follow-up of the entire cohort was 15 months (range 3–99 months) and the median follow-up of surviving patients was 38 months (range 9–99 months). In total, 26 patients (43.33%) suffered a relapse (local relapse *n* = 3, lymph nodes and/or distant metastases *n* = 15, unclear metastasis pattern *n* = 8). Of the 33 (55.00%) patients who died during the follow-up, 26 (43.33%) of them died due to BLCA.

### 2.2. ANLN and TLE2 as Risk Markers for Prognostic Prediction After RC

**Figure 1:**
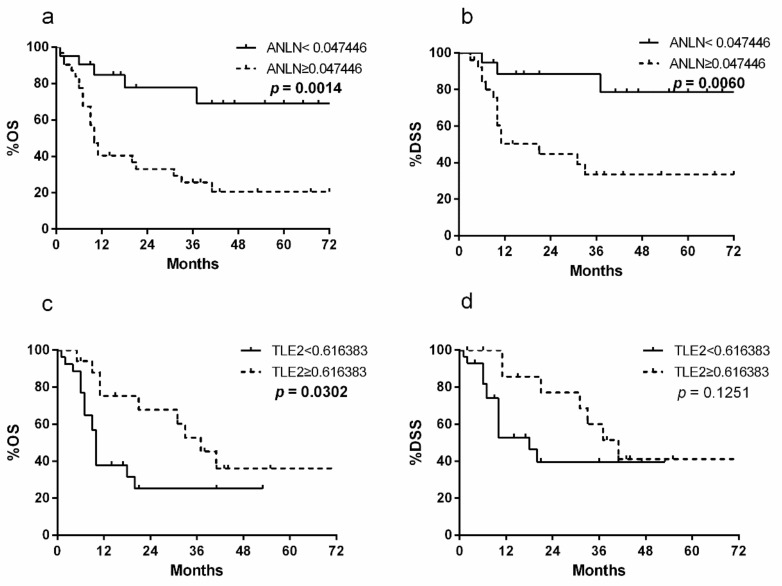
Kaplan–Meier plots of overall- (OS) and disease-specific (DSS) survival associated with a*nilin actin binding protein* (*ANLN*) and t*ransducing-like enhancer protein 2* (*TLE2*) risk stratification in the Mannheim cohort. The group with high *ANLN* expression showed worse OS (**a**) and DSS (**b**) than the group with low expression. The group with high *TLE2* expression displayed more favorable OS (**c**) and DSS (**d**) than the group with low expression.

In the Mannheim cohort, patients with high *ANLN* expression showed worse overall survival (OS) (*n* = 22 with low expression, *n* = 38 with high expression; median survival, 21 vs. 10 months, and *p* = 0.0010) and disease-specific survival (DSS) (*n* = 20 with low expression, *n* = 40 with high expression; median survival, 21 vs. 10 months, and *p* = 0.0060) after RC than patients with low expression (Figure 1a,b). Conversely, patients with high *TLE2* expression displayed more favorable OS (*n* = 29 with high expression, *n* = 31 with low expression; median survival, 15 vs. 9 months, and *p* = 0.0236) and DSS (*n* = 29 with high expression, *n* = 31 with low expression; median survival, 31 vs. 13 months, and *p* = 0.2083) than patients with low expression (Figure 1c,d). The gene expression levels were not normally distributed.

In the univariate and multivariate Cox regression analysis, *ANLN* (*p* = 0.0020 and *p* = 0.0390, respectively) and *TLE2* (*p* = 0.0120 and *p* = 0.0020, respectively) expression were independent predictors. Furthermore, lymph node status was identified as an independent prognostic factor by both analyses (*p* = 0.0030 and *p* = 0.0240), as was lymphovascular invasion (LVI) (*p* = 0.0040) by only the univariate Cox regression analysis (Table 2). No significant correlation was observed between patient age, patient gender, or stage of tumor and *ANLN* and *TLE2* expression.

**Figure 2:**
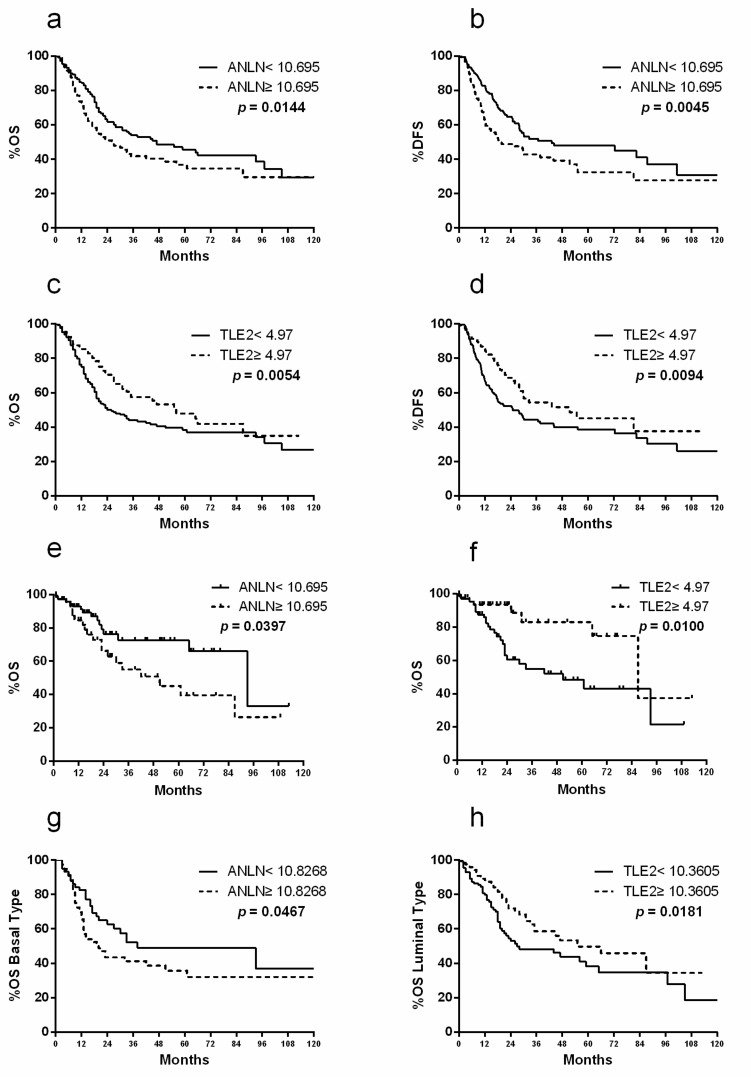
Kaplan–Meier plots of OS and disease-free survival (DFS) associated with *ANLN* and *TLE2* risk stratification in The Cancer Genome Atlas (TCGA) cohort. Higher *ANLN* expression showed worse OS (**a**) and DFS (**b**). Higher *TLE2* expression showed more favorable OS (**c**) and DFS (**d**). In the T2 subgroup, low *ANLN* (**e**) and *TLE2* (**f**) expression showed good and poor prognosis of OS, respectively. Higher *ANLN* expression showed worse OS in basal subtype (**g**) and higher *TLE2* showed better OS in luminal subtype (**h**).

In The Cancer Genome Atlas (TCGA) cohort, higher *ANLN* expression was associated with worse OS (*n* = 246 with low expression, *n* = 161 with high expression; median survival, 18.07 vs. 15.31 months, and *p* = 0.0144) and disease-free survival (DFS) (*n* = 246 with low expression, *n* = 161 with high expression; median survival, 18.82 vs. 13.99 months, and *p* = 0.0045, Figure 2a,b). In contrast, higher *TLE2* expression was associated with more favorable OS (*n* = 250 with low expression, *n* = 157 with high expression; median survival, 16.69 vs. 18.99 months, and *p* = 0.0054) and DFS (*n* = 250 with low expression, *n* = 157 with high expression; median survival, 15.31 vs. 16.79 months, and *p* = 0.0094, Figure 2c,d). In the TCGA cohort, correlation between *ANLN* and *TLE2* expression and stage showed that *ANLN* was significantly expressed in pT3 and pT4 (higher stages, median expression 9.95 vs. 10.16, and *p* = 0.0109, Figure S1a), while *TLE2* was dominantly expressed in pT2 (lower stage, median expression 9.88 vs. 9.73, and *p* = 0.0228, Figure S1b). Further analysis of the TCGA bladder cancer cohort according to clinical stage showed that lower *ANLN* (*n* = 77 with low expression, *n* = 41 with high expression; median survival, 23.43 vs. 19.66 months, and *p* = 0.0397) and *TLE2* (*n* = 67 with low expression, *n* = 51 with high expression; median survival, 17.94 vs. 20.37 months, and *p* = 0.0100) expression in the pT2 subgroup could be attributed to a good and poor prognosis of OS, respectively (Figure 2e,f). Furthermore, the gene expression of *ANLN* and *TLE2* was analyzed in subtypes of MIBC. Notably, in the TCGA cohort, *ANLN* expression in patients with MIBC and basal subtype was associated with worse OS (*n* = 61 with low expression, *n* = 81 with high expression; median survival, 20.37 vs. 13.96 months, and *p* = 0.0467, Figure 2g) compared with the whole TCGA cohort. Higher *TLE2* expression showed better OS in patients with MIBC and luminal subtype (*n* = 130 with high expression, *n* = 115 with low expression; median survival, 19.48 vs. 17.87 months, and *p* = 0.0181, Figure 2h) compared with the whole TCGA cohort. The gene expression levels were not normally distributed.

In the univariate Cox regression analysis of clinicopathological features, stage (*p* < 0.001), lymph node status (*p* < 0.001), *ANLN* (*p* = 0.0160), and *TLE2* (*p* = 0.0060) expression were independent. Furthermore, stage (*p* = 0.0060), *ANLN* (*p* = 0.0180), and *TLE2* (*p* = 0.0400) were also independent prognostic factors in the multivariate Cox regression analysis (Table 3).

### 2.3. Gene Expression Profiling of ANLN and TLE2 in Comparison with other Tumor Entities

Bar plots compared the gene expression profiles across all 31 tumor samples and paired normal tissues from TCGA database. *ANLN* expression was confirmed to be up-regulated in the majority of tumor samples (26/31, 83.87%), while *TLE2* was down-regulated in the majority of tumor samples (25/31, 80.64%). In BLCA tumor samples, *ANLN* expression was up-regulated (median expression 16.72) compared to normal tissues (median expression 1.54; Figure S2a), while *TLE2* expression was down-regulated (median expression 22.27) compared to normal tissues (median expression 60.99; Figure S2b). The dot-box plots with data normalized for log-scale showed that *ANLN* expression was significantly higher in BLCA tumor samples than normal tissues (median expression 4.15 vs. 1.34, and *p* < 0.001, Figure S2c). In contrast, *TLE2* expression was significantly lower in BLCA samples than in normal tissues (median expression 4.54 vs. 5.95, and *p* < 0.001, Figure S2d).

### 2.4. Association of Copy-number Alterations with ANLN and TLE2 Gene Expression

**Figure 3:**
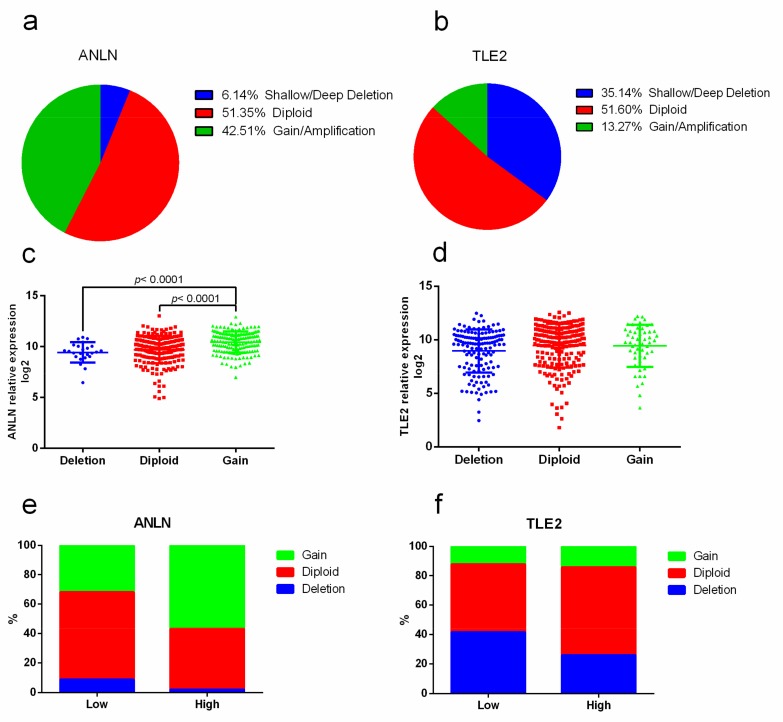
*ANLN* and *TLE2* expression in association with copy-number alterations. *ANLN* showed 6.14% of shallow/deep deletion, 51.35% of diploid, and 42.51% of gain/amplification (**a**). *TLE2* showed 35.14% of shallow/deep deletion, 51.60% of diploid and 13.27% of gain/amplification (**b**). Significant higher expression of *ANLN* was observed in the subgroup with gain than diploid (median expression 10.54 vs. 9.88, and *p* < 0.0001) and deletion (median expression 10.54 vs. 9.45, and *p* < 0.0001) (**c**). No significant differences of *TLE2* expression were found in the subgroups with deletion against diploid (median expression 9.63 vs. 9.98, and *p* = 0.1458) or gain (median expression 9.63 vs. 9.73, and *p* = 0.9190) (**d**). The gene expression levels of *ANLN* and *TLE2* were grouped into high and low expression. In the subgroup with lower *ANLN* expression, diploid (*n* = 143, 59.34%) was more frequently observed than gain (*n* = 77, 31.95%) and deletion (*n* = 21, 8.71%). The subgroup with higher *ANLN* expression is associated with a higher percentage of gain (*n* = 86, 56.90%) than diploid (*n* = 62, 41.10%) and deletion (*n* = 3, 2.00%) (**e**). *TLE2* was mainly expressed in the subgroup with deletion and diploid in *TLE2* low expression (*n* = 102, 41.46% in deletion; *n* = 114, 46.34% in diploid) and *TLE2* high expression (*n* = 40, 25.97% in deletion; *n* = 92, 59.74% in diploid). Gain was observed in only 12.2% (*n* = 30) in *TLE2* low expression group and in 14.29% (*n* = 22) in the *TLE2* high expression group (**f**).

Analysis of *ANLN* and *TLE2* gene expression in association with copy-number alterations revealed a genetic alteration rate of 10% (41/407) for *ANLN* and 5% (21/407) for *TLE2*. Putative copy-number alterations including deep/shallow deletion, diploid, gain, and amplification were acquired from GISTIC (Genomic Identification of Significant Targets in Cancer). The majority of copy-number alteration signatures for *ANLN* were gains and amplifications rather than deletions (number 173 vs. 25, 42.51% vs. 6.14%, median expression 10.54 vs. 9.45, and *p* = 0.0002, Figure 3a). For *TLE2*, most samples showed deletion variations rather than amplifications (number 143 vs. 54, 35.14% vs. 13.27%, median expression 9.63 vs. 9.77, and *p* = 0.0394, Figure 3b). Significantly higher expression of *ANLN* was observed in the subgroup with gain compared to diploid (median expression 10.54 vs. 9.88, and *p* < 0.0001) and deletion (median expression 10.54 vs. 9.45, and *p* < 0.0001, Figure 3c). There were no significant differences in *TLE2* expression in the subgroups with deletion compared to diploid (median expression 9.63 vs. 9.98, and *p* = 0.1458) or gain (median expression 9.63 vs. 9.73, and *p* = 0.9190, Figure 3d). When gene expression levels were grouped into low and high expression, analysis revealed higher rates of amplification/gains in the subgroup with higher *ANLN* expression (*n* = 86, 56.90%) compared to lower *ANLN* expression (*n* = 77, 31.95%, Figure 3e). In contrast, amplification was only observed in 12.2% (*n* = 30) of the *TLE2* low expression group and in 14.29% (*n* = 22) of the *TLE2* high expression group (Figure 3f). Furthermore, higher rates of deletion were seen in the *TLE2* low expression subgroup than in the *TLE2* high expression subgroup (*n* = 102, 41.46% vs. *n* = 40, 25.97%).

### 2.5. Correlation with Signaling Pathways and Therapeutic Targets in Bladder Cancer

The protein-protein interactions of *ANLN* and *TLE2* were analyzed by Search Tool for Retrieval of Interacting Genes/Proteins (STRING, Figure S3). The number of counted gene sets and the false discovery rate for each Gene Ontology (GO) term are shown in Table S2. The interaction network based on curated databases and experimentally derived results showed that key molecules in signaling pathways were significantly correlated with *ANLN* and *TLE2*, including cell proliferation (false discovery rate, FDR = 1.99^−10^), Notch signaling (FDR = 2.38^−9^), Wnt signaling (FDR = 2.38^−9^), and hormone receptor (FDR = 3.16^−8^). The correlation with important therapeutic targets in BLCA, including *epidermal growth factor receptor* (*EGFR)* (*p* = 7.73^−31^ for *ANLN* and *p* = 1.78^−14^ for *TLE2*), *Erb-B2 receptor tyrosine kinase 2* (*ERBB2)* (*p* = 3.44^−13^ for *ANLN* and *p* = 1.96^−43^ for *TLE2*), *fibroblast growth factor receptor 3* (*FGFR3)* (*p* = 6.52^−9^ for *ANLN* and *p* = 0.0016 for *TLE2*), and *programmed death-ligand 1* (*PD-L1)* (*p* = 2.89^−18^ for *ANLN* and *p* = 1.07^−18^ for *TLE2*) were found to be significantly correlated with *ANLN* and *TLE2* based on TCGA data (Table 4).

It is noteworthy that *ANLN* was positively correlated with cell proliferation markers including *cyclin-dependent kinase 1* (*CDK1)* (ρ = 0.594, *p* = 3.98^−40^), *Rac GTPase activating protein 1* (*RACGAP1)* (ρ = 0.725, *p* = 1.41^−67^), *marker of proliferation Ki-67* (*MKI67)* (ρ = 0.711, *p* = 7.56^−64^), and *forkhead box M1* (*FOXM1)* (ρ = 0.688, *p* = 2.98^−58^). In contrast, *TLE2* was negatively correlated with cell proliferation molecules involving *CDK1* (ρ = -0.338, *p* = 2.63^−12^), *RACGAP1* (ρ = -0.451, *p* = 9.46^−22^), *MKI67* (ρ = -0.396, *p* = 1.07^−16^), and *FOXM1* (ρ = -0.400, *p* = 4.46^−17^). *TLE2* was also correlated with molecules involved in Wnt signaling including *catenin beta 1* (*CTNNB1)* (ρ = -0.276, *p* = 1.6^−8^) and hormone receptors including *forkhead box A1* (*FOXA1)* (ρ = 0.505, *p* = 9.11^−28^) and *GATA binding protein 3* (*GATA3)* (ρ = 0.65, *p* = 2.8^−50^; Table 4). Further, expression of *RACGAP1*, *MKI67*, *FOXM1*, *CDK1*, *CTNNB1*, and *GATA3* were examined in the Mannheim cohort. This analysis showed that *ANLN* was significantly correlated with *RACGAP1* (ρ = 0.455, *p* < 0.0001), *FOXM1* (ρ = 0.549, *p* < 0.0001), *MKI67* (ρ = 0.577, *p* < 0.0001), and *CDK1* (ρ = 0.763, *p* < 0.0001; Figure S4a–d). *TLE2* was significantly correlated with *GATA3* (ρ = 0.409, *p* = 0.0012) and *CTNNB1* (ρ = 0.363, *p* = 0.0070) (Figure S4e,f). Similar expression data was also detected in the urothelial cell lines (Figure S5a).

### 2.6. Molecular Subtype Specificity of ANLN and TLE2

In silico RNA-seq data from the Cancer Cell Line Encyclopedia showed *ANLN* and *TLE2* expression levels in TPM (transcripts per million) for 25 BLCA cell lines, with different molecular subtypes of each (basal, luminal, and mixed; Figure S5b). Cell lines that were classified as basal subtypes, including UMUC3 and SCaBER, showed slightly higher expression of *ANLN*, while *TLE2* dominantly expressed in cell lines classified as luminal subtypes, e.g., RT112 and RT4. qPCR based on SYBR Green showed relative expression of *ANLN* and *TLE2* in five malignant urothelial cell lines (RT4, RT112, UMUC3, T24, and ScaBER), which correspond with the RNA-seq data (Figure S5a). Similarly, in patients with BLCA (TCGA, Provisional), expression of *ANLN* was higher in the basal than in the luminal subtypes (median expression 10.93; range 6.1–13.01 vs. median expression 9.64; range 4.87–12.25, *p* < 0.0001, Figure S5c) according to the mRNA clustering. In contrast, *TLE2* showed an opposite trend with a higher expression in luminal than basal subtypes (median expression 10.41; range 5.04–12.55 vs. median expression 7.41, range 1.79–12.25, *p* < 0.0001, Figure S5d). In addition, the basal subtype marker *KRT5* and the luminal subtype marker *KRT20* were analyzed in the Mannheim cohort. *KRT5* was significantly correlated with *ANLN* (ρ = 0.278, *p* = 0.042; Figure S6a), and *KRT20* was significantly correlated with *TLE2* (ρ = 0.296, *p* = 0.026; Figure S6b). The expression of *ANLN* and *TLE2* were also analyzed in the high *KRT5* and the high *KRT20* expression group, which were classified based on median expression of *KRT5* and *KRT20*. It was observed that *ANLN* expression was higher in the high *KRT5* group than in the high *KRT20* group (*p* = 0.1119, Figure S6c), and *TLE2* expression was higher in the high *KRT20* group than in the high *KRT5* group (*p* = 0.1413, Figure S6d).

## 3. Discussion

This study aimed to retrospectively evaluate the prognostic and clinical impact of *ANLN* and *TLE2* gene expression and to validate these results in published datasets. In order to evaluate the translational benefit, gene expression was compared with molecular subtypes, targets, and relevant clinicopathologic parameters in multivariable analyses. Higher *ANLN* transcript levels were found to be associated with worse OS and DSS in the Mannheim cohort, which corresponds with results from previous studies of BLCA and upper urinary tract urothelial carcinoma [23]. Together, these results indicate that *ANLN*, in addition to lymph node status, may be an independent predictor for progression-free survival (PFS), and superior to the use of T stage and LVI as predictors. As a key regulator of cytokinesis, it is not surprising that *ANLN* might play a critical role in carcinogenesis [24]. Previous research has established that knockdown of *ANLN* could significantly inhibit the proliferation of bladder cancer both in vitro and in vivo. Furthermore, knockdown of *ANLN* strongly suppressed the migration and invasion ability of J82 and 5637 bladder cancer cell lines [16]. Additionally, in upper urinary tract urothelial carcinoma, overexpression of *ANLN* in the nucleus is a poor prognostic factor, which was confirmed by data on protein levels, while low expression of *ANLN* in the cytoplasm is a poor prognosis maker [23].

In contrast to *ANLN*, *TLE2* was found to be dominantly expressed in patients with lower stages of BLCA. Patients with higher *TLE2* expression had a more favorable OS and DFS, both in the whole-stage group and in the T2 subgroup. These results, together with univariate and multivariate Cox regression analysis of the Mannheim and the TCGA cohort, suggest that *TLE2* could serve as an independent risk factor for prognostic prediction of patients with BLCA. The most recent research about *TLE2* showed an inhibition of replication and transcription in Kaposi’s sarcoma-associated herpesvirus [19]. To our knowledge, there is only very limited data about the role of *TLE2* in cancer and no data specific to BLCA. Therefore, our findings are the first describing the role of *TLE2* in BLCA. In adition, the Kaplan–Meier curves of ANLN and TLE2 with OS and DSS showed separation at earlier time points, but convergence at later time points, between the high and low expression group in the TCGA cohort. It indicated that the predictive capabilities of *ANLN* and *TLE2* expression might be stronger at earlier time points after RC.

In our study, *ANLN* expression was strongly correlated with cell proliferation markers including *CDK1*, *RACGAP1*, *MKI67*, and *FOXM1*. In urothelial carcinoma cell lines, these genes showed a consistently strong positive correlation with the expression of *ANLN*, which is highly expressed in ScaBER cells and expressed in low amounts in RT4 cells. In our patient cohort, we also found a strong correlation of *ANLN* with *RACGAP1*, *MKI67*, and *CDK1*. These results indicate that *ANLN* could have a crucial role in tumor proliferation of MIBC. It is already known that the proliferation markers *MKI67* and *RACGAP1* have a significant importance in BLCA [25,26]. Previous research showed that mRNA expression of *MKI67* is significantly correlated and associated with stage and grade in NMIBC [27]. Furthermore, the expression of *RACGAP1* correlated significantly with the tumor stage in BLCA after RC, and *RACGAP1* was strongly expressed in the early stages of NMIBC patient samples [28]. Additionally, *FOXM1* has been shown to be overexpressed on the mRNA and protein levels in bladder cancer cells, and plays an important role in cisplatin resistance, outcome prediction, and risk stratification of patients with BLCA [29].

In this study, *TLE2* expression correlated with several Wnt pathway components, such as β-catenin (CTNNB1), *TCF7,* and *LEF1* in the TCGA cohort. By analyzing the expression in the Mannheim cohort, it was also found that *TLE2* was significantly correlated with *CTNNB1*. In addition, *CTNNB1* is highly expressed in RT4 cells and expressed in lower amounts in ScaBER cells, which is similar to the expression pattern of *TLE2*. The activation of the Wnt/β-catenin signaling pathway plays an important role in tumorigenesis and development of various cancers including BLCA [30,31,32]. Gain-of-function mutations in *CTNNB1* are detected in numerous human cancers [33,34,35]; therefore, it is necessary to explore the role of Wnt/β-catenin regulated genes in BLCA. Previous research has shown that *TLE2* was significantly suppressed and the level of β-catenin protein was increased in esophageal tumor cells, both of which were modulated by NDRG1 overexpression [36]. Interestingly, *ANLN* positively correlates with *CTNNB1*, while *TLE2* correlates inversely with *CTNNB1,* which was also observed in the Mannheim cohort. Although there is no direct correlation between *ANLN* and *TLE2* in both cohorts, the involvement of *ANLN* and *TLE2* underlying the Wnt/β-catenin signaling pathway was preliminarily revealed by our study.

To reveal more details of *ANLN* and *TLE2* expression in bladder cancer, the RNA-seq data of transcripts based on TCGA cohort were analyzed. Interestingly, the splice variants of *ANLN* and *TLE2* showed different expression levels, with *ANLN*-201 (ENST00000265748.6), *ANLN*-202 (ENST00000396068.6), *ANLN*-210 (ENST00000457743.1), *ANLN*-212 (ENST00000491782.1), *TLE2*-201 (ENST00000262953.10), *TLE2*-202 (ENST00000426948.6), *TLE2*-204 (ENST00000455444.6), and *TLE2*-215 (ENST00000590101.5) highly expressed compared with other transcripts. These findings indicate that differential expression patterns of *ANLN* and *TLE2* splice variants might potentially have a practical usefulness, which needs further investigations.

Our study demonstrates that *ANLN* and *TLE2* show a distinct subtype-specific overexpression in BLCA cell lines. *ANLN* showed overexpression in basal-like urothelial carcinoma cell lines and patients, while *TLE2* showed significantly higher expression in luminal-like urothelial carcinoma cell lines and patients. Interestingly, for patients with MIBC in the whole TCGA cohort, *ANLN* and *TLE2* harbored similar prognostic values for basal and luminal subtypes, respectively. Molecular classification has emerged as a promising research tool beyond histopathology to stratify cancer patients for personalized medicine [37]. Recent years have witnessed increasing interest and research into the molecular basis of bladder cancer [6]. Several studies subclassified both MIBC and NMIBC through RNA-seq-based data and identified distinct molecular subtypes that correlated well with outcome and therapy response [5,38,39]. MIBC molecular subtypes, which are broadly grouped into basal and luminal subtypes, showed similarities to the molecular phenotypes of breast cancer [40]. It is believed that epithelial-to-mesenchymal transition (EMT) is a critical step in the progression of breast cancer, particularly the basal-like one [41]. The basal and EMT/claudin-low markers were highly expressed in the same subtype of patients with BLCA based on the TCGA database [42]. Due to the significant association with basal subtype of BLCA, it is suggested that *ANLN* could play a potential role in EMT in BLCA. Also, *KRT5* is highly upregulated in basal and *KRT20* in luminal subtype in BLCA [6]. qPCR-based molecular subtyping of BLCA by *KRT5* and *KRT20* mRNA expression is a method associated with the survival of patients with MIBC [43]. It is confirmed that *KRT5* and *KRT20* showed significant association with *ANLN* and *TLE2* in the Mannheim cohort. The differences of *ANLN* and *TLE2* expression were not significant in the high *KRT5* and *KRT20* group maybe due the fact that only KRT5 and KRT20 were used for subtype association.

Until recently, effective targets following platinum-based chemotherapy were limited for patients with advanced urothelial carcinoma. The most promising option is immunotherapy with programmed cell death 1 (PD-1)/programmed cell death ligand 1 (PD-L1) checkpoint inhibitors, coupled with the anti-cytotoxic T-lymphocyte-associated protein 4 (CTLA-4) antibodies [44,45]. Apart from differences in expression thresholds for defining PD-L1 positivity, the validated biomarkers for optimal patient selection are still unrevealed [46]. In our study, expression of *ANLN* and *TLE2* correlate with the therapeutic targets for bladder cancer as indicated in existing research and clinical trials. Notably, *ANLN* was positively and *TLE2* was negatively correlated with *PD-L1* in the TCGA cohort. *TLE2* also showed a negative correlation with *PD-1* and the key immunoregulator *CTLA-4*. These results suggest that *ANLN* and *TLE2* could serve as potential biomarkers for response to immunotherapy and precise therapeutic management of MIBC.

## 4. Materials and Methods

### 4.1. Patients and Tissue Samples

This study retrospectively enrolled sixty patients who received RC at the Department of Urology and Urosurgery of the University Medical Centre Mannheim between 2008 and 2011 and who had a histological diagnosis of MIBC (*n* = 47, 78% male, median age: 72 years, range: 41–87 years; *n* = 13, 22% females, median age: 74 years, and range: 71–86 years; Mannheim cohort). All patients were treated with RC and bilateral lymphadenectomy without preoperative or adjuvant chemotherapy or radiotherapy. With the help of the clinic’s internal documentation program, the following parameters were collected after examination of the pathology findings: sex, age, T-stage, N-stage, M-stage, grading, lymphovascular invasion (LVI), blood vessel invasion (VI), simultaneous carcinoma in situ (CIS), multifocality, and soft tissue positive surgical margin.

Formalin fixed paraffin embedded (FFPE) tumor tissue samples were evaluated for pathological stage according to the 2017 TNM classification from the Union for International Cancer Control (UICC) [47]. Tumors were graded using the 2017 WHO/ISUP classification [48] (Table 1). Studies involving human participants were approved by the ethical board of University Medical Centre Mannheim (2015-549-MA) and performed in accordance with relevant guidelines and regulations. The Cancer Genome Atlas cohort (TCGA, Provisional) contained RNA sequencing data of 407 patients with MIBC and complete clinicopathological data and follow-up data.

### 4.2. Database

Expression data of 25 human bladder cancer cell lines were collected from Cancer Cell Line Encyclopedia (Novartis/Broad, Nature 2012), including 20 bladder urothelial cell carcinomas, one bladder squamous cell carcinoma, and four bladder carcinoma cell lines from unknown primaries. Expression of *ANLN* and *TLE2* were analyzed by Expression Atlas (https://www.ebi.ac.uk/gxa/home) and normalized by transcripts per million (TPM). Expression data of 9736 tumor samples and 8,587 normal samples across 31 types of tissues were collected from TCGA (https://tcga-data.nci.nih.gov/tcga/) and the Genotype-Tissue Expression (GTEx) projects (https://gtexportal.org/) and analyzed by cBioPortal (http://www.cbioportal.org/) and Gene Expression Profiling Interactive Analysis (GEPIA, http://gepia.cancer-pku.cn) [49]. The height of a bar in the resulting bar plots represents the median expression tumor types or normal tissue. Each dot represents the expression of *ANLN* and *TLE2* in samples in the dot-box plots. The protein-protein interactions were analyzed by STRING (https://string-db.org/). GO enrichment analysis and Kyoto Encyclopedia of Genes and Genomes (KEGG) pathways were analyzed by Enrichr (http://amp.pharm.mssm.edu/Enrichr/) [50]. Expression of transcripts and exons was analyzed by Xena (https://xena.ucsc.edu/).

### 4.3. Cell Lines

Six different cell lines were used in this study, including one normal human urothelium cell line (UROtsa), two basal-like urothelial carcinoma cell lines (ScaBER and UMUC3), two luminal-like urothelial carcinoma cell lines (RT112 and RT4), and one mixed-type urothelial carcinoma cell line (T24). UROtsa cells were cultured in Roswell Park Memorial Institute medium (RPMI) with 5% fetal bovine serum (FBS). RT112, RT4, ScaBER, and UMUC3 cells were cultured in Dulbecco’s modified Eagle’s medium (DMEM) containing 10% FBS. T24 cells were cultured in McCoy’s 5A medium containing 10% FBS. UMUC3, ScaBER, RT112 and T24 cells were obtained from the European Collection of Authenticated Cell Cultures (ECACC), RT4 from the American Type Culture Collection (ATCC), and UROtsa cells from a collaborator. All cells were incubated at 37 °C in an atmosphere of 5% CO_2_, per manufacturer instructions. Before starting the experiment, all cell lines were authenticated by Multiplexion (Heidelberg, Germany).

### 4.4. RNA Extraction and qRT-PCR

The total RNA of all cell lines was isolated using the RNeasy Mini kits (Qiagen, Hilden, Germany) according to the manufacturer’s instructions using a high salt buffer content and binding of RNA to a silica membrane. After washing steps, contaminants were removed and then RNA was eluted in RNase-free water. For FFPE tissues, RNA was extracted and enriched using the magnetic-bead-based XTRAKT FFPE Kit (Stratifyer, Cologne, Germany) according to the instructions of manufacturer [28]. Next, reverse transcription was performed for cell samples using the M-MLV Reverse Transcriptase kit (Invitrogen, Thermo Fisher Scientific, Waltham, MA, USA), and, for FFPE samples, using the Superscript III^®^ reverse transcriptase kit (Invitrogen, Thermo Fisher Scientific, Waltham, MA, USA) with sequence-specific primers. qPCR was used to measure relative mRNA expression with TaqMan Fast advanced Master Mix (Invitrogen, Thermo Fisher Scientific, Waltham, MA, USA). Experiments were performed on a StepOnePlus (Applied Biosystems, Darmstadt, Germany) with 20 s at 95 °C, followed by 40 cycles of 3 s at 95 °C, and 30 s at 60 °C. β-Glucuronidase (GUS) and Calmodulin2 (Calm2) were measured as reference genes [51,52]. The relative mRNA expression level was normalized to reference genes and determined using the 2^−ΔΔCT^ method for cell culture samples and the 40-∆CT for FFPE samples, as previously described [53]. All primers and probes used in this study are shown in Supplementary Table S1.

### 4.5. Statistical Analysis

Statistical analyses were performed with SPSS 20.0 (IBM, Chicago, IL, USA) and GraphPad Prism 6.0 (GraphPad Software, La Jolla, CA, USA). A Kolmogorov–Smirnov (K-S) test was used to determine whether the data were normally distributed. Student’s *t*-tests were used to compare between groups of normally distributed numerical data, while Mann–Whitney U and Kruskal–Wallis tests were used to compare the non-normally distributed numerical data. Linear regression was used to determine the efficiency of amplification. Spearman tests were used to test the correlation between different gene expressions. The cut-off values (2^−ΔΔCT^ value) of the high (≥0.047466) and low (<0.047466) *ANLN* expression groups, as well as the high (≥0.616383) and low (<0.616383) *TLE2* expression groups were determined by receiver operating characteristic (ROC) curve analysis in the Mannheim cohort [54]. Similarly, in the TCGA cohort, the cut-off value (log2 value) of high (≥10.695 in the whole group and ≥10.8268 in the basal subgroup) and low (<10.695 in the whole group and <10.8268 in the basal subgroup) *ANLN* expression group, as well as high (≥4.97 in the whole group and ≥10.3605 in the luminal subgroup) and low (<4.97 in the whole group and <10.3605 in the luminal subgroup) *TLE2* expression group was determined by ROC curve analysis. The Cox regression model was used for univariate and multivariate analysis to calculate hazard ratio (HR). Survival rates of patients were calculated by the Kaplan–Meier method, and comparison was made by the log-rank test. In all cases, *p* < 0.05 was considered statistically significant.

## 5. Conclusions

This study provides in silico and in vitro evidence supporting the prognostic potential of *ANLN* and *TLE2* for patients with MIBC. These results indicate that developing *ANLN* and *TLE2* as new biomarkers will help to further optimize personalized therapy for these patients.

## Figures and Tables

**Table 1:** Clinicopathological characteristics of patients and specimens of the Mannheim cohort.

Clinicopathological Features		*n*
Age	<70	23
	≥70	37
Gender	Male	47
	Female	13
Grade	Low	5
	High	55
Stage	T1	6
	T2	11
	T3	28
	T4	15
Lymph node metastasis	Negative	37
	Positive	15

**Table 2:** Univariate and multivariate Cox regression analysis of *ANLN* and *TLE2* with clinicopathological features in the Mannheim cohort (HR = hazard ratio, CI = confidence interval, LVI = lymphovascular invasion, significant *p* Values are bold).

Factor	Univariate		Multivariate	
	HR (95% CI)	*p* Value	HR (95% CI)	*p* Value
Diagnosis Age				
<70 vs. ≥70	0.584 (0.269–1.269)	0.174	−	−
Gender				
Male vs. Female	1.128 (0.405–3.140)	0.957	−	−
Stage				
T1/T2 vs. T3/4	0.168 (0.015–1.832)	0.128	−	−
LVI				
Negative vs. Positive	0.542 (0.359–0.819)	**0.004**	−	−
Lymph node Statues				
Negative vs. Positive	0.549 (0.371–0.813)	**0.003**	0.612 (0.399–0.938)	**0.024**
*ANLN*				
Low vs. High	0.220 (0.084–0.575)	**0.002**	0.328 (0.114–0.945)	**0.039**
*TLE2*				
Low vs. High	0.305 (0.121–0.769)	**0.012**	0.172 (0.057–0.519)	**0.002**

**Table 3:** Univariate and multivariate Cox regression analysis of *ANLN* and *TLE2* with clinicopathological features in the TCGA cohort (HR = hazard ratio, CI = confidence interval, significant *p* Values are bold).

Factor	Univariate		Multivariate	
	HR (95% CI)	*p* Value	HR (95% CI)	*p* Value
Diagnosis Age				
<70 vs. ≥70	1.260 (0.931–1.705)	0.134	−	−
Gender				
Male vs. Female	1.257 (0.902–1.751)	0.177	−	−
Smoking Status				
No vs. Yes	1.335 (0.940–1.897)	0.106	−	−
Stage				
T2 vs. T3/4	1.950 (1.393–2.731)	**<0.001**	1.646 (1.156–2.342)	**0.006**
Lymph node Statues				
Negative vs. Positive	2.145 (1.596–2.883)	**<0.001**	1.989 (1.461–2.707)	**<0.001**
*ANLN*				
Low vs. High	1.439 (1.070–1.934)	**0.016**	1.438 (1.064–1.943)	**0.018**
*TLE2*				
Low vs. High	0.636 (0.460–0.880)	**0.006**	1.415 (1.015–1.973)	**0.040**

**Table 4:** Correlation of *ANLN* and *TLE2* with key molecules in signaling pathways and therapeutic targets (correlation coefficient values above 0.4 and below −0.4 are bold).

Correlated Gene		ANLN		TLE2	
		Correlation Coefficient	*p* Value	Correlation Coefficient	*p* Value
Cell proliferation	CDK1	**0.594**	3.98 × 10^−40^	−0.338	2.63 × 10^−12^
	RACGAP1	**0.725**	1.41 × 10^−67^	**−0.451**	9.46 × 10^−22^
	MKI67	**0.711**	7.56 × 10^−64^	−0.396	1.07 × 10^−16^
	FOXM1	**0.688**	2.98 × 10^−58^	**−0.4**	4.46 × 10^−17^
Notch signaling	NOTCH1	0.109	0.027294	−0.19	0.000115
	RBPJ	−0.196	6.6 × 10^−5^	0.05	0.311468
Wnt signaling	TCF7	0.067	0.175097	0.006	0.89689
	TCF7L1	0.162	0.001066	−0.267	4.75 × 10^−8^
	TCF7L2	−0.186	0.000164	0.23	2.84 × 10^−6^
	LEF1	−0.101	0.041347	−0.026	0.596286
	CTNNB1	0.237	1.29 × 10^−6^	−0.276	1.6 × 10^−8^
Hormone receptor signaling	AR	−0.185	0.000171	0.388	4.9 × 10^−16^
	ESR1	−0.003	0.944815	−0.048	0.332592
	ESR2	−0.191	0.00011	0.334	4.33 × 10^−12^
	FOXA1	−0.38	1.89 × 10^−15^	**0.505**	9.11 × 10^−28^
	GATA3	**−0.403**	2.33 × 10^−17^	**0.65**	2.80 × 10^−50^
Therapeutic targets	EGFR	**0.53**	7.73 × 10^−31^	−0.368	1.78 × 10^−14^
	ERBB2	−0.35	3.44 × 10^−13^	**0.613**	1.96 × 10^−43^
	FGFR3	−0.283	6.52 × 10^−9^	0.156	0.001575
	PIK3CA	**0.426**	2.19 × 10^−19^	−0.264	6.34 × 10^−8^
	CDK4	0.207	2.68 × 10^−5^	−0.267	4.69 × 10^−8^
	HRAS	−0.023	0.648147	−0.183	0.000202
	PDCD1	0.0881	0.0754	−0.313	1.07 × 10^−10^
	PD-L1	**0.414**	2.89 × 10^−18^	**−0.419**	1.07 × 10^−18^
	CTLA4	0.0963	0.052	−0.363	3.71 × 10^−14^
	EZH2	**0.446**	2.51 × 10^−21^	−0.162	0.001009

## Supplementary Materials

The following are available online at https://www.mdpi.com/2072-6694/11/12/1840/s1, Table S1: Primers and probes used in this study, Table S2: GO enrichment analysis by Enrichr indicated the significant GO terms for *ANLN* and *TLE2* including biological process, molecular function, cellular component, and KEGG pathways, Figure S1: Correlation between *ANLN* and *TLE2* expression and stage from the TCGA bladder cancer cohort (TCGA, Provisional) showed that *ANLN* was significantly expressed in higher T3-4 stages (median expression 9.95 vs. 10.16, *p* = 0.0109) (a), while *TLE2* was dominantly expressed in lower T2 stage (median expression 9.88 vs. 9.73, *p* = 0.0228) (b), Figure S2: Expression of *ANLN* and *TLE2* in BLCA tumor samples compared with normal tissues. ANLN expression was up-regulated (median expression 16.72) in tumors compared with normal tissues (median expression 1.54) based on TCGA data (a). *TLE2* expression was down-regulated in tumors (median expression 22.27) compared with normal tissues (median expression 60.99) based on TCGA data (b). *ANLN* expression was significantly higher in BLCA tumor samples than normal tissues (median expression 4.15 vs. 1.34, *p* < 0.001) based on TCGA and GTEx projects (c), while *TLE2* expression was significantly lower in in BLCA tumor samples than normal tissues (median expression 4.54 vs. 5.95, *p* < 0.001) based on TCGA and GTEx projects (d), Figure S3: Protein-protein interactions predicted by STRING showed the interaction network of *ANLN* (a) and *TLE2* (b) based on curated databases and experimental determination. Figure S4: Correlation of selected genes in the Mannheim cohort. *ANLN* was significantly correlated with RACGAP1 (a), FOXM1 (b), MKI67 (c), and CDK1 (d). *TLE2* was significantly correlated with GATA3 (e) and CTNNB1 (f), Figure S5: qPCR showed relative expression of ANLN and TLE2 in five malignant urothelial cell lines (RT4, RT112, UMUC3, T24, and ScaBER) (a). In silico RNA-seq data from the Cancer Cell Line Encyclopedia showed expression level in TPM (transcripts per million) for *ANLN* and *TLE2* in 25 BLCA cell lines with different molecular subtypes of each (b). Expression of *ANLN* was higher in basal than luminal subtype (median expression 10.93 with range of 6.1 to 13.01 vs. median expression 9.64 with range of 4.87 to 12.25, p < 0.0001) in patients with BLCA (c). *TLE2* expression was higher in luminal than basal subtype (median expression 10.41 with range of 5.04 to 12.55 vs. median expression 7.41 with range of 1.79 to 12.25, p < 0.0001) (d), Figure S6: Correlation of *KRT5* and *ANLN* (a) and *KRT20* and *TLE2* (b) in the Mannheim cohort. *ANLN* expression was higher in the high *KRT5* group than in the high *KRT20* group (*p* = 0.1119, (c), and *TLE2* expression was higher in the high *KRT20* group than in the high *KRT5* group (*p* = 0.1413, d).
