# Supplementary material for: ANLN and TLE2 in Muscle Invasive Bladder Cancer: A Functional and Clinical Evaluation Based on In Silico and In Vitro Data
Source: Cancers (Basel). 2019 Nov 21;11(12):1840. doi: 10.3390/cancers11121840 (PMC6966660; doi:10.3390/cancers11121840)
Supplement: Supplementary file 1 [file cancers-11-01840-s001.pdf]

Supplementary Materials

ANLN and *TLE2* in Muscle Invasive Bladder Cancer: A Functional and Clinical Evaluation Based on In Silico and In Vitro Data

Sheng Wu, Katja Nitschke, Jakob Heinkele, Cleo-Aron Weis, Thomas Stefan Worst, Markus Eckstein, Stefan Porubsky and Philipp Erben

Table S1. Primers and probes used in this study.

Gene	Forward primer Sequence (5' → 3')	Reverse primer Sequence (5' → 3')	Probe Sequence (5' label → 3' label)
ANLN	GCATTAGCAGAAAGCAGCGA	TGGACACTAAACTCTCTGGACT	FAM-GAACAGGAAGATGCACTGAATATCTCCTCAATGTCTTTA-BHQ1
Calm2	GAGCGAGCTGAGTGGTTGTG	AGTCAGTTGGTCAGCCATGCT	VIC-TCGCGTCTCGGAAACCGGTAGC-BHQ1
CDK1	AAAACTACAGGTCAAGTGGTAGCCAT	GCATAAGCACATCCTGAAGACTGACT	FAM-TCAGACTAGAAAGTGAAGAGGAAGGGGTTCCTAGTACTG-BHQ1
CTNNB1	GGGTCCTCTGTGAACTTGCTC	TTCTTGTAATCTTGTGGCTTGTCC	FAM-ACAAGGAAGCTGCAGAAGCTATTGAAGCTGAGG-BHQ1
FOXM1	GACCACCTGGAGCCCTTTG	GATGTTGGATAGGCTATTGTTGATAGTG	FAM-CGAGCAGAAACGGGAGACCTGTGATGGTGAGG-BHQ1
GATA3	GCAATGCCTGTGGGCTCTAC	TTCTGGTCTGGATGCCTTCCT	FAM-ACAAGCTTCACAATATTAACAGACCCCTGACTATGAAG-BBQ
GUS	GAAAATAYRTGGTTGGAGAGCTCATT	CCGAGTGAAGATCCCCTTTTTA	VIC-CCAGCACTCTCGTCGGTGACTGTTCA-BHQ1
KRT5	CGCCACTTACCGCAAGCT	ACAGAGATGTTGACTGGTCCAACTC	FAM-TGGAGGGCGAGGAATGCAGACTCA-BBQ
KRT20	GCGACTACAGTGCATATTACAGACAA	CACACCGAGCATTTTGCAGTT	FAM-TTGAAGAGCTGCGAAGTCAGATTAAGGATGCT-BBQ
MKI67	TGCTACTCCAAAGAAGCCTGTG	GTATGAGCTTTCCCTATTATTATGGTAC	FAM-CGAAGTTCACAGTCAATTTAGTACAGGCCAC-TAM
RacGap1	GAATGTGCGGAATCTGTTTGAG	TCGCCAACTGGATAAATTGGA	FAM-ACTGAGAATCTCCACCCGGCGCA-BHQ2
TLE2	AAGCGTCTGAGCGGTATCTG	TGCTGCTGCCCGATGAG	FAM-GCTCAGATTATCCCCTTCCTGACCCAGGAGCAT-BHQ1

Table S2. GO enrichment analysis by Enrichr indicated the significant GO terms for *ANLN* and *TLE2* including biological process, molecular function, cellular component, and KEGG pathways.

Gene	Dataset	Pathway ID	Pathway Description	Count in Gene Set	False Discovery Rate
ANLN	Biological Process (GO)	GO:0007067	mitotic nuclear division	10	1.42×10^{-13}
		GO:0051301	cell division	9	1.99×10^{-10}
		GO:1903047	mitotic cell cycle process	9	7.57×10^{-9}
		GO:0000278	mitotic cell cycle	9	1.69×10^{-8}
		GO:0051302	regulation of cell division	7	2.14×10^{-8}
	Molecular Function (GO)	GO:0005524	ATP binding	6	0.0483
		GO:0043168	anion binding	7	0.0483
	Cellular Component (GO)	GO:0005819	spindle	7	3.16×10^{-8}
		GO:0044430	cytoskeletal part	9	4.71×10^{-7}
		GO:0015630	microtubule cytoskeleton	8	1.33×10^{-6}
		GO:0030496	midbody	5	1.33×10^{-6}
		GO:0072686	mitotic spindle	4	1.64×10^{-6}
TLE2	Biological Process (GO)	GO:0000122	negative regulation of transcription from RNA polymerase II promoter	9	2.39×10^{-8}
		GO:0043588	skin development	7	2.39×10^{-8}
		GO:0048864	stem cell development	7	2.93×10^{-8}
		GO:2000736	regulation of stem cell differentiation	6	3.28×10^{-8}
		GO:0009790	embryo development	9	5.72×10^{-8}
	Molecular Function (GO)	GO:0003700	transcription factor activity, sequence-specific DNA binding	9	7.79×10^{-8}
		GO:0044212	transcription regulatory region DNA binding	8	7.79×10^{-8}
		GO:0003682	chromatin binding	7	5.90×10^{-7}
		GO:0001047	core promoter binding	5	2.16×10^{-6}
		GO:0070491	repressing transcription factor binding	4	4.10×10^{-6}
	Cellular Component (GO)	GO:0005667	transcription factor complex	7	3.16×10^{-8}
		GO:0005654	nucleoplasm	11	1.54×10^{-7}
		GO:0031981	nuclear lumen	10	3.92×10^{-5}
		GO:0070369	beta-catenin-TCF7L2 complex	2	8.60×10^{-5}

	GO:0002193	MAML1-RBP-Jkappa- ICN1 complex	2	0.000129
KEGG Pathways	05216	Thyroid cancer	5	3.57×10^{-10}
	04310	Wnt signaling pathway	6	2.38×10^{-9}
	04330	Notch signaling pathway	5	2.38×10^{-9}
	05213	Endometrial cancer	5	2.38×10^{-9}
	05200	Pathways in cancer	7	2.75×10^{-9}

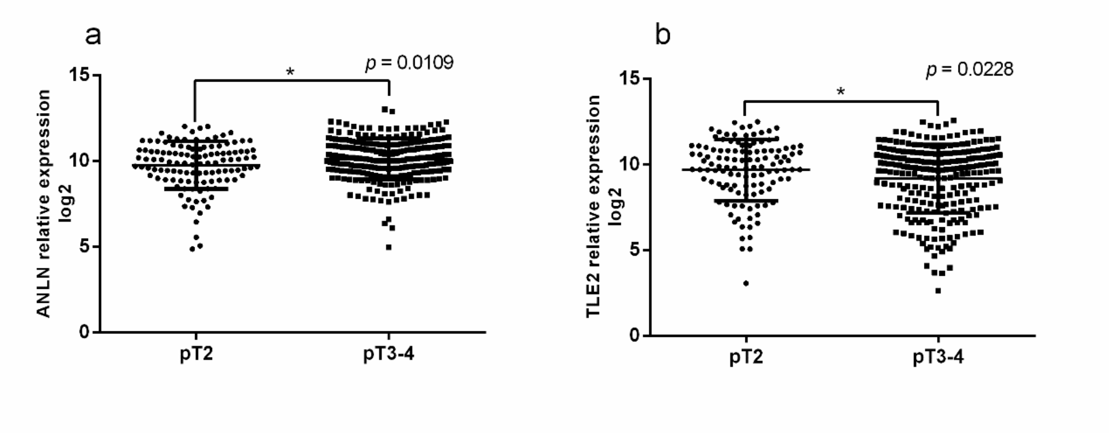

Figure S1. Correlation between *ANLN* and *TLE2* expression and stage from the TCGA bladder cancer cohort (TCGA, Provisional) showed that *ANLN* was significantly expressed in higher T3-4 stages (median expression 9.95 vs. 10.16, $p = 0.0109$) (**a**), while *TLE2* was dominantly expressed in lower T2 stage (median expression 9.88 vs. 9.73, $p = 0.0228$) (**b**).

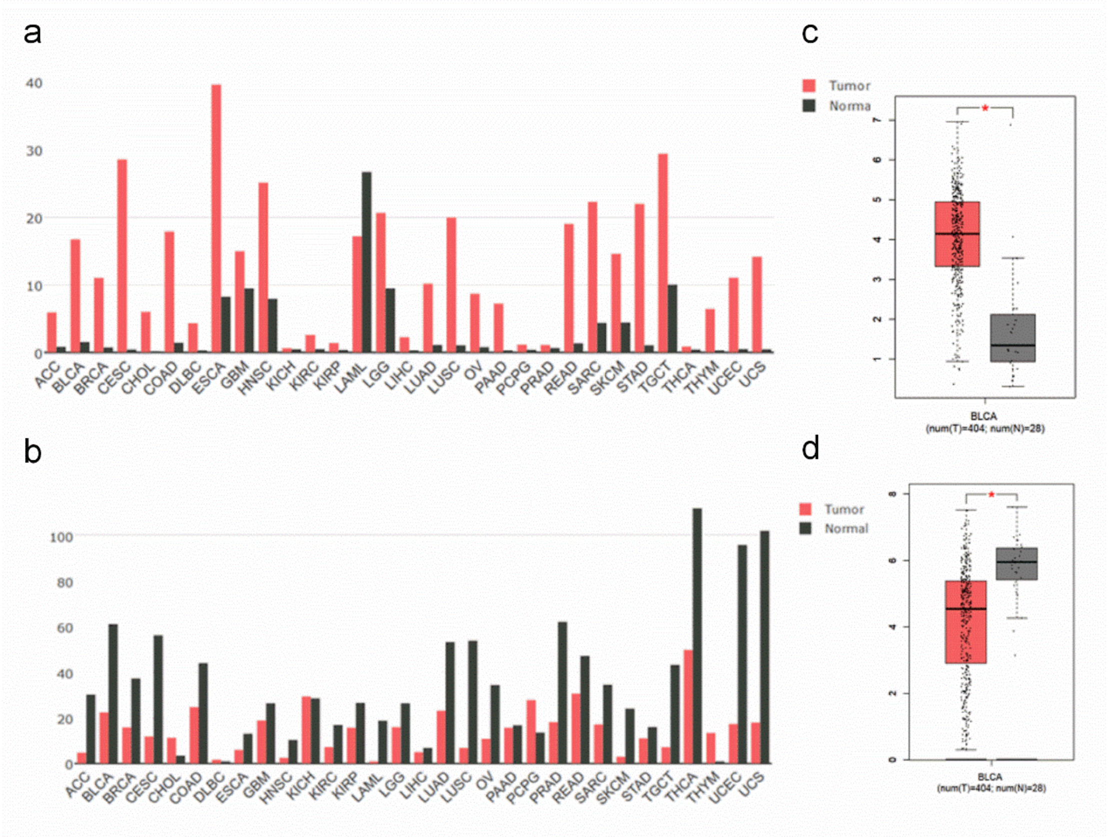

Figure S2. Expression of *ANLN* and *TLE2* in BLCA tumor samples compared with normal tissues. *ANLN* expression was up-regulated (median expression 16.72) in tumors compared with normal tissues (median expression 1.54) based on TCGA data (**a**). *TLE2* expression was down-regulated in tumors (median expression 22.27) compared with normal tissues (median expression 60.99) based on TCGA data (**b**). *ANLN* expression was significantly higher in BLCA tumor samples than normal tissues (median expression 4.15 vs. 1.34, $p < 0.001$) based on TCGA and GTEx projects (**c**), while *TLE2* expression was significantly lower in in BLCA tumor samples than normal tissues (median expression 4.54 vs. 5.95, $p < 0.001$) based on TCGA and GTEx projects (**d**).

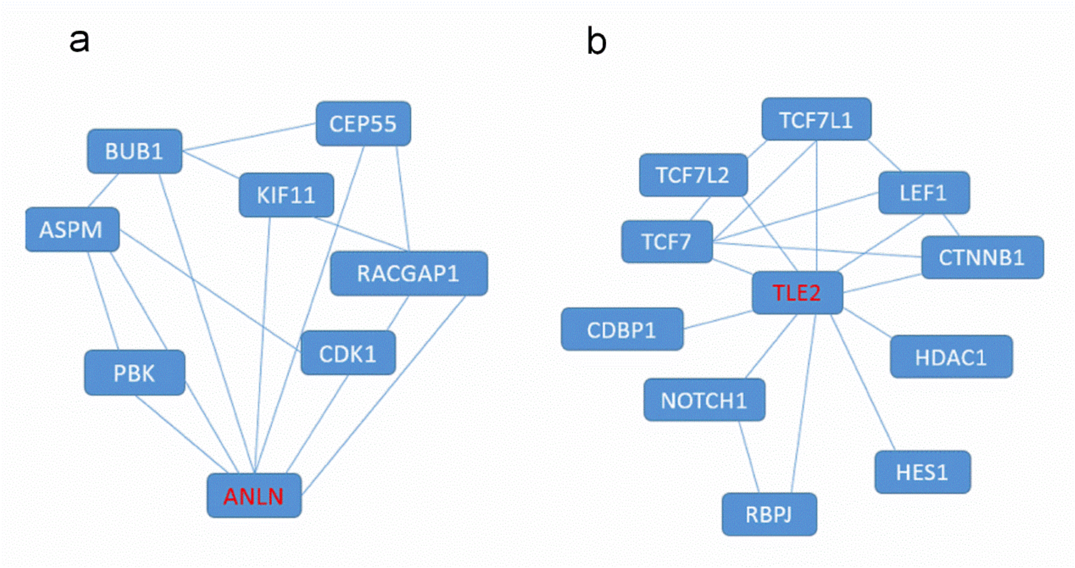

Figure S3. Protein-protein interactions predicted by STRING showed the interaction network of *ANLN* (**a**) and *TLE2* (**b**) based on curated databases and experimental determination.

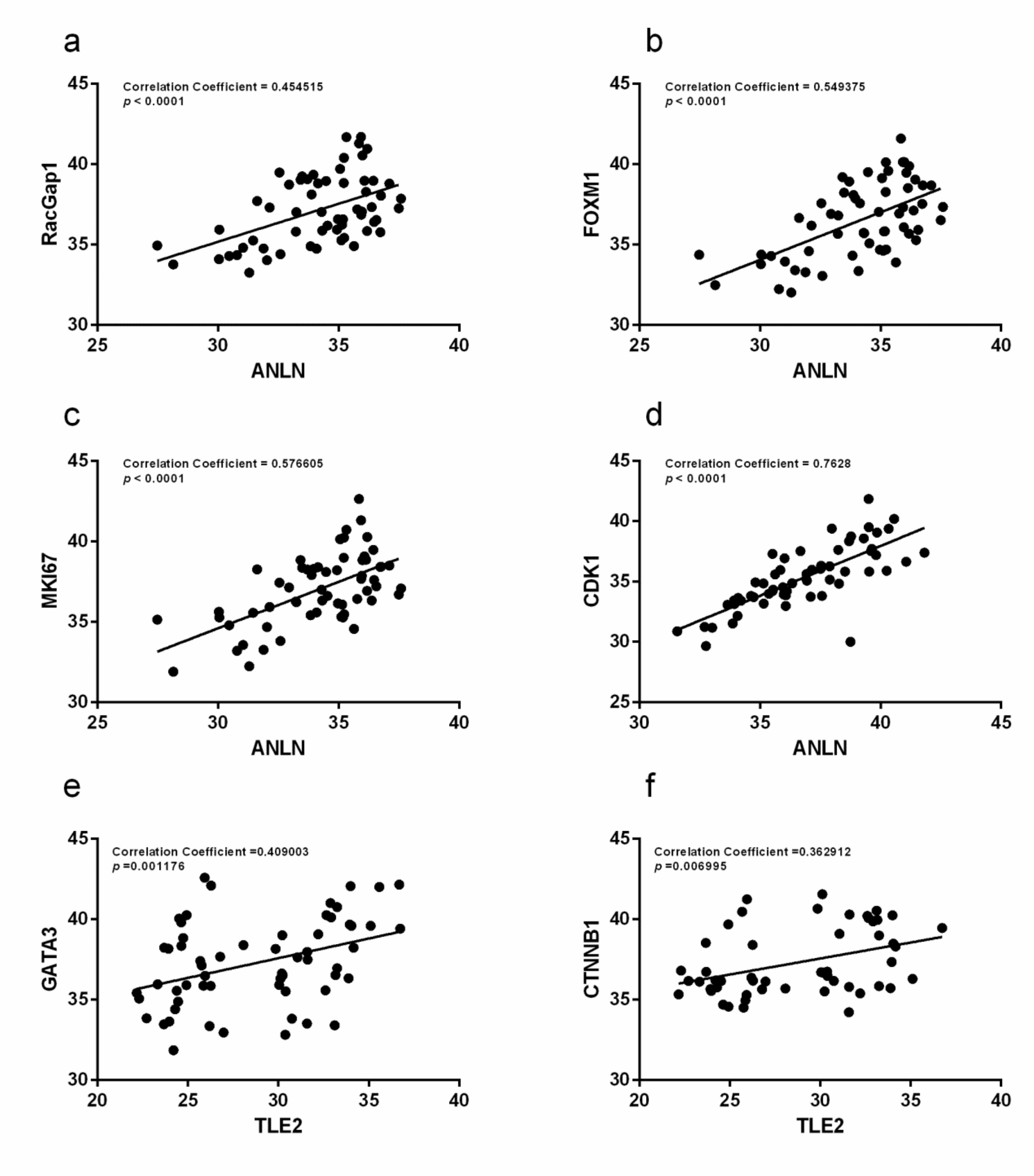

Figure S4. Correlation of selected genes in the Mannheim cohort. *ANLN* was significantly correlated with RACGAP1 (**a**), FOXM1 (**b**), MKI67 (**c**), and CDK1 (**d**). *TLE2* was significantly correlated with GATA3 (**e**) and CTNNB1 (**f**).

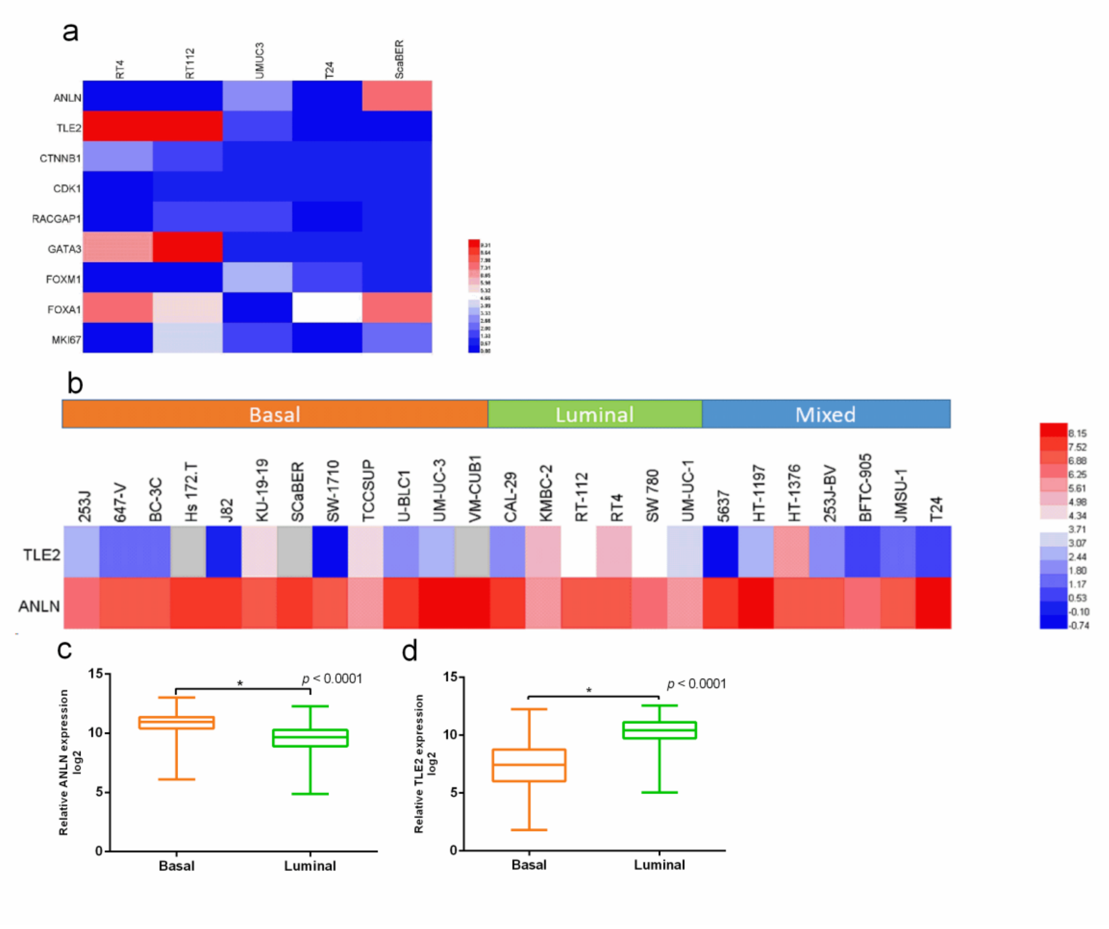

Figure S5. qPCR showed relative expression of *ANLN* and *TLE2* in five malignant urothelial cell lines (RT4, RT112, UMUC3, T24, and ScaBER) (**a**). *In silico* RNA-seq data from the Cancer Cell Line Encyclopedia showed expression level in TPM (transcripts per million) for *ANLN* and *TLE2* in 25 BLCA cell lines with different molecular subtypes of each (**b**). Expression of *ANLN* was higher in basal than luminal subtype (median expression 10.93 with range of 6.1 to 13.01 vs. median expression 9.64 with range of 4.87 to 12.25, $p < 0.0001$) in patients with BLCA (**c**). *TLE2* expression was higher in luminal than basal subtype (median expression 10.41 with range of 5.04 to 12.55 vs. median expression 7.41 with range of 1.79 to 12.25, $p < 0.0001$) (**d**).

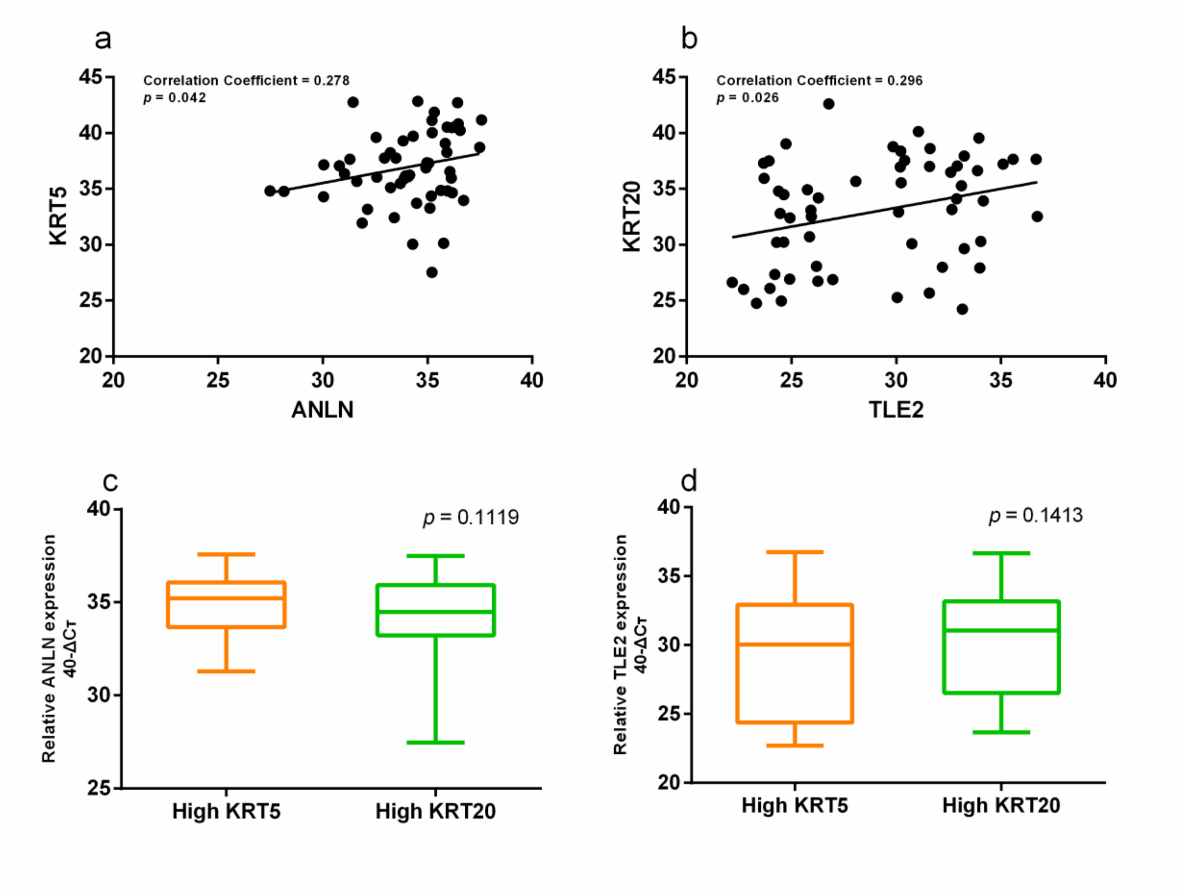

Figure S6. Correlation of *KRT5* and *ANLN* (**a**) and *KRT20* and *TLE2* (**b**) in the Mannheim cohort. *ANLN* expression was higher in the high *KRT5* group than in the high *KRT20* group ($p = 0.1119$, (**c**), and *TLE2* expression was higher in the high *KRT20* group than in the high *KRT5* group ($p = 0.1413$, (**d**).

© 2019 by the authors. Licensee MDPI, Basel, Switzerland. This article is an open access article distributed under the terms and conditions of the Creative Commons Attribution (CC BY) license (http://creativecommons.org/licenses/by/4.0/).
